# Bimetallic Ti_2_NbC_2_ MXene as anode material for metal ion batteries: influence of functional groups

**DOI:** 10.1039/d5ra04549e

**Published:** 2025-08-26

**Authors:** Rodrigo Ponce Perez, Jonathan Guerrero Sanchez, Maria G. Moreno Armenta

**Affiliations:** a Centro de Nanociencias y Nanotecnología, Universidad Nacional Autónoma de México Ensenada B.C CP 22800 Mexico rponce@ens.cnyn.unam.mx moreno@ens.cnyn.unam.mx

## Abstract

By DFT calculations, we investigate the effect of functional groups on the electrochemical activity of the Ti_2_NbC_2_ MXene as an anode in alkali and alkaline earth batteries. Our findings show that the H3 site is the most favorable adsorption site for O, Cl, F, and OH surface functionalization. The electronic properties of functionalized MXenes are investigated, finding a metallic behavior in all cases. The Li, Na, K, Ca, and Mg intercalation process was evaluated in the functionalized MXenes by systematically inserting atoms. The electrochemical properties are investigated by calculating the open-circuit voltage as a function of theoretical gravimetric capacities. Results demonstrate that Ti_2_NbC_2_(OH)_2_ is unfavorable for energy storage applications. At the same time, Cl- and F-functionalized MXenes provide the lower theoretical gravimetric capacities of less than 100 mAh g^−1^ for alkali metal ions and are unstable for alkaline earth elements. On the other hand, Ti_2_NbC_2_O_2_ MXene shows an excellent performance. The theoretical gravimetric capacities for Li, Na, and Mg ions are 274, 219, and 438 mAh g^−1^, respectively. Similar values to those reported for low atomic weight MXenes, evidencing their capacity to store metal ions. Our findings demonstrate the capacity of oxidized Ti_2_NbC_2_ to be implemented in energy storage devices.

## Abstract 2

By DFT calculations, we investigate the effect of functional groups on the electrochemical activity of the Ti_2_NbC_2_ MXene as an anode in alkali and alkaline earth batteries.

## Graphical abstract

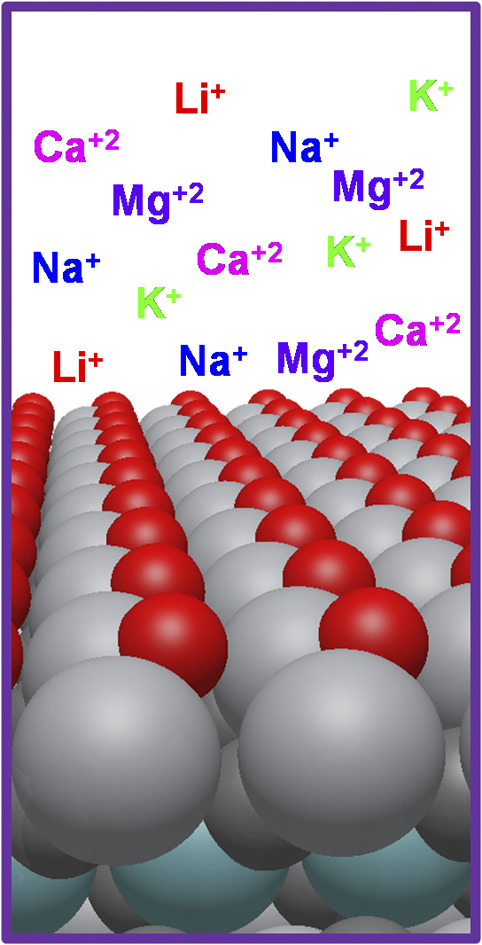

## 1. Introduction

Since the discovery of MXenes in 2011 by the group of Prof. Gogotsi,^1^ in which they proposed the existence of Ti_3_C_2_T_*z*_, a tremendous collaborative effort has been made in advancing the knowledge and applications of such a 2D family. The versatility of MXenes is vast and related to many possible structures comprising the MAX phases, with more than 300 materials.^2^ Once the MAX phases are etched, usually employing HF, it is possible to obtain MXenes, which have a general formula M_*n*+1_X_*n*_T_*x*,_ where M is a transition metal, X stands for C or N, and T defines the surface passivation; the common functional groups are O, OH, and F.^3^ However, HF-free synthesis, for example, employing molten salts, can lead to Cl terminations.^4^ That is not it; MXenes also exist with randomly distributed atoms or layered alloys^5^ and recently as high-entropy MXenes,^6^ and well-defined in-plane disorder (i-MXenes),^7^ all with tunned properties. Numerous reviews have been published discussing the impact of MXenes in chemistry and physics, as well as their applications in various fields, including electronics, catalysis, photocatalysis, sensors, energy storage, and environmental applications.^8–12^ In this manuscript, we focus on an important one: ion storage.

Until now, graphite has been the primary anode material used in commercial Li-ion batteries (LIBs), with a theoretical gravimetric capacity of 372 mAh g^−1^.^13^ Although graphite is the most commercially used, it has a low theoretical gravimetric capacity, especially compared to the theoretical gravimetric capacity of silicon (4200 mAh g^−1^) at low potentials.^14^ Still, silicon anodes have rapid degradation due to the significant volumetric expansion of around 400% when Li enters the lattice. It also generates an enormous strain, which induces electrode cracking into small particles and poor conductivity.^14^

Although graphite is well-established in the market, the benchmark for new-generation anodes of around 1000 mAh g^−1^ is still far from being reached.^15^ Then, new generation anode materials appeared in the literature to establish a benchmark. In this sense, MXenes have emerged as promising materials. For example, V_2_C with a controlled interlayer distance using Co atoms reaches a superior capacity of 1117.3 mAh g^−1^ at 0.1 A g^−1^ with enormous cycling stability of around 15 000 cycles.^16^ The eptalayer V_4_C_3_ MXene delivers a 225 mAh g^−1^ capacity after 300 cycles at 0.1 A g^−1^.^17^ A specific capacity lower than the Co modified V_2_C MXene. Also, the molten salt-derived Nb_2_CT_*x*_ MXene can reach a capacity of 330 mAh g^−1^ at 0.05 A g^−1^, which surpasses the capacity achieved by Ti_3_C_2_T_*x*_ (205 mAh g^−1^) obtained by the same method.^18^ On the other hand, doping with N in the C layer enables a 288 mAh g^−1^ capacity to be reached after 1500 cycles.^19^ Therefore, modifying MXenes by intercalating atoms that modulate their interlayer distance and doping is an effective tool for engineering their gravimetric capacity. Furthermore, combining MXenes in diverse setups with other 3D and 2D materials delivers capacities that range from 100 mAh g^−1^ for 2D MoS_2_/Ti_3_C_2_ to 2118 mAh g^−1^ for Si/MXene, see ref. 20 and 21 and the references therein.

Recently, Liu *et al.* reported the successful synthesis of the bimetallic Ti_2_NbC_2_ MXenes.^22^ Additionally, they investigated their use as an anode in LIBs, finding a specific capacity of 196.2 mAh g^−1^, a high retention capacity of 100% after 400 cycles, and an 81% retention after 4000 cycles. To our knowledge, there is no information at the atomic scale of the intercalation process and its use in alkali and alkaline earth batteries. Therefore, in this work, we considered the double transition metal Ti_2_NbC_2_T_*x*_ MXenes as potential anodes for ion storage (Li, Na, K, Mg, and Ca). Our findings suggest that oxidized MXenes are the most promising candidates for use in energy storage. Results show a theoretical gravimetric capacity of 273, 219, 109, 219, and 438 mAh g^−1^, employing Li, Na, K, Ca, and Mg atoms. The manuscript is organized as follows: Section 2 is for methodology, Section 3 is for the results, and Section 4 contains the conclusions.

## 2. Methodology

Li, Na, K, Ca, and Mg intercalation into bimetallic Ti_2_NbC_2_T_*x*_ MXene (with T_*z*_ = O, Cl, F, and OH) is investigated through first-principles calculations. The calculations are done within the periodic density functional theory (DFT) as implemented in the *Vienna Ab initio Simulation Package* (VASP) code.^23–25^ The exchange–correlation energy is treated according to the generalized gradient approximation (GGA) with Perdew–Burke–Ernzerhof (PBE) parametrization,^26^ which has demonstrated accurate results in similar systems.^27,28^ In the calculations, van der Waals interactions are considered by using the DFT-D3 method of Grimme with a zero-damping function.^29^ The projected augmented wave (PAW) pseudopotentials^30,31^ are considered to treat the electron–ion interactions with 450 eV as the energy cutoff. To investigate the intercalation process, we employed the supercell method. Each supercell is formed by an MXene monolayer in a p(2 × 2) periodicity and a vacuum space larger than 15 Å to avoid interactions between periodic layers. In geometry optimization, convergence is achieved when the energy differences and force components are less than 1 × 10^−4^ eV and 0.01 eV Å^−1^, respectively. The Brillouin zone is sampled with a *k*-point mesh of 8 × 8 × 1 according to the Monkhorst–Pack scheme^32^ for the primitive unit cell and adapted according to the periodicity of the supercell. The metal ion diffusion is investigated by performing CI-NEB calculations^33,34^ with seven intermediate images. For more details, see Fig. S1 from the SI.

## 3. Results and discussion

### 3.1 Ti_2_NbC_2_T_*x*_ MXene

**Fig. 1:**
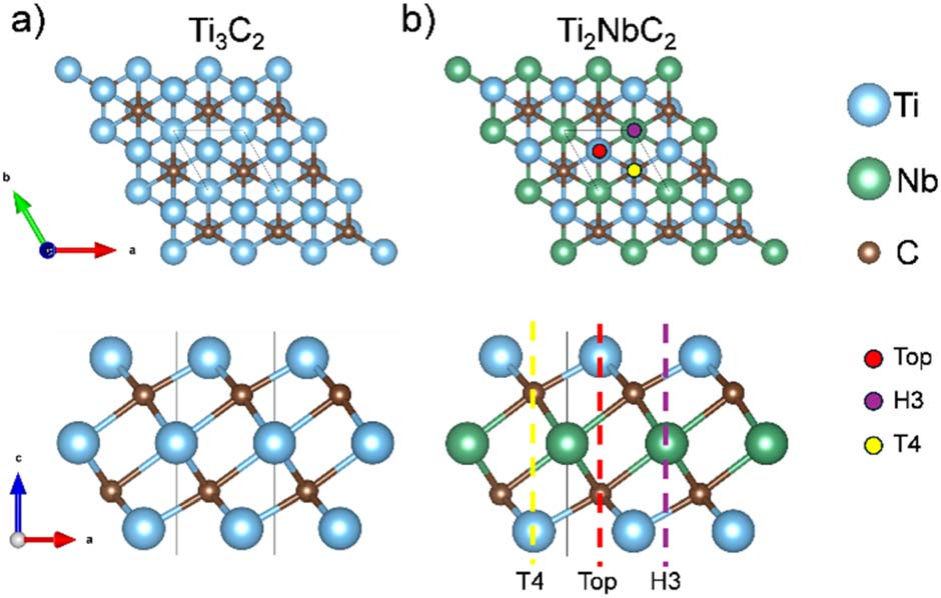
Top and side views of (a) Ti_3_C_2_ MXene and (b) Ti_2_NbC_2_ MXene.

The Bare Ti_3_C_2_ MXene is a penta-layer formed by alternate Ti/C/Ti/C/Ti monolayers with A/B/C/A/B stacking with optimized cell parameter *a* = 3.07 Å and a layer thickness of 4.64 Å. On the other hand, pristine Ti_2_NbC_2_ has a central layer of Nb atoms replacing the Ti sites. The calculated cell parameter is 3.11 Å with a layer thickness of 4.74 Å. Fig. 1a and b show the side and top views of both MXenes. However, MXenes can be obtained in an A/B/A/B/A stacking,^35^ see Fig. S2. Therefore, we calculated the energy difference between both stackings, defined as: Δ*E* = *E*^A/B/C/A/B^ − *E*^A/B/A/B/A^. The first and second terms of the right side of the equation represent the energy of the MXene with the A/B/C/A/B and A/B/A/B/A stacking, respectively. Results show a value of −2.41 and −2.14 eV for the Ti_3_C_2_ and Ti_2_NbC_2_, respectively, demonstrating the high stability of the A/B/C/A/B stacking. Therefore, only consider this phase in this work. To investigate the surface functionalization of the Ti_2_NbC_2_ MXene, we considered three different high symmetry sites denoted as Top, T4, and H3 – see Fig. 1b. The Top site is on top of the most exposed atom, T4 is on top of the atoms of the second layer, in this case, on top of the C atoms, and the H3 site is a hollow site located on top of the central layer. We also considered the following functional groups: Cl, F, O, and OH. The adsorption energy is calculated using the following equation:1
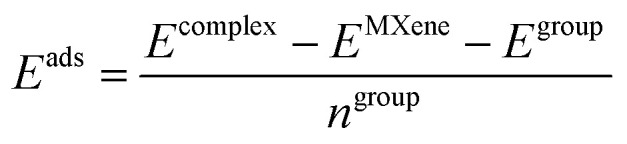


Where *E*^complex^ is the total energy of the system at hand, *E*^MXene^ is the energy of the bare MXene, *E*^group^ is the energy of the isolated functional group, and *n*^group^ is the number of functional groups present in the cell. The isolated functional groups (Cl, F, O, and OH) were modeled in an empty box of 15 Å in length. Table 1 summarizes the results. Notice that we have negative values, suggesting that adsorption is favorable. Besides, the H3 site provides the lowest energy values, with oxygen providing the most intense adsorption (−8.34 eV/functional group), followed by F (−6.72 eV/functional group), OH (−5.59 eV/functional group), and Cl (−5.07 eV/functional group). Additionally, surface functionalization with F and OH groups does not significantly alter the cell parameter; however, O functionalization results in a contraction, while Cl functionalization leads to an expansion. Bader charge analysis evidence that oxygen accepts 1.11*e* from Ti, which implies a strong interaction between them, promoting the contraction in the cell parameter. On the other hand, Cl only accepts 0.61*e* from Ti; this could be related to the ion size, which reduces their interaction and expands the cell parameter.

**Table 1:** Adsorption energies (in eV/functional group), optimized cell parameters for the most stable configuration, and Bader charge of the functional group

Functional group	*E* ^ads^ (eV/functional group) high symmetry site			Optimized cell parameter (Å)	Bader charge (*e*)
	H3	T4	Top		
O	−8.38	−7.57	−5.94	3.05	1.11
Cl	−5.07	−4.78	−4.16	3.20	0.61
F	−6.72	−6.34	−6.01	3.10	0.75
OH	−5.59	−5.33	−4.69	3.10	0.75

**Fig. 2:**
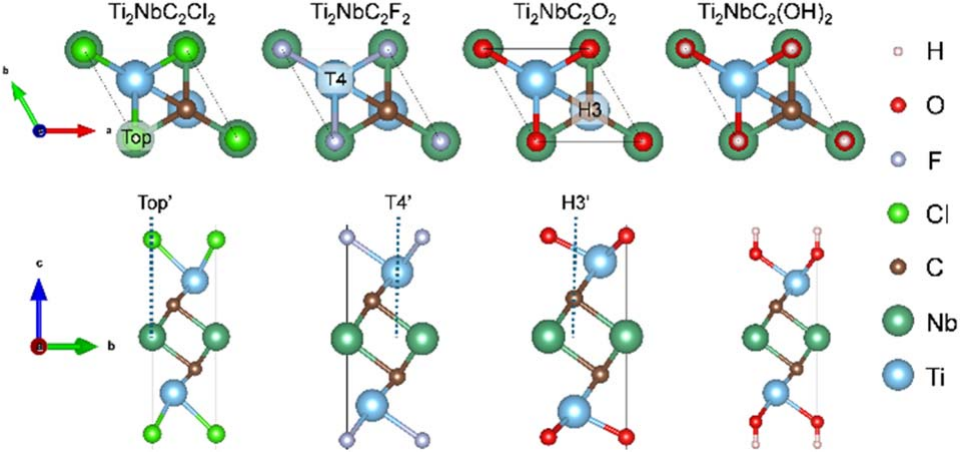
Top and side views of the functionalized MXenes considered in this work. The high symmetry sites Top, T4, and H3 are shown.

Fig. 2 shows the atomistic structure of the functionalized systems. The Cl-functionalized MXene has a bond distance Cl–Ti of 2.52 Å and a thickness of 8.04 Å. The Ti_2_NbC_2_F_2_ provides an F–Ti bond distance of 2.18 Å and a layer thickness of 7.28 Å, about the Ti_2_NbC_2_O_2_ (Ti_2_NbC_2_(OH)_2_) MXene; the O–Ti bond distance is 1.98 Å (2.20 Å) with a layer thickness of 7.05 Å (9.32 Å).

**Fig. 3:**
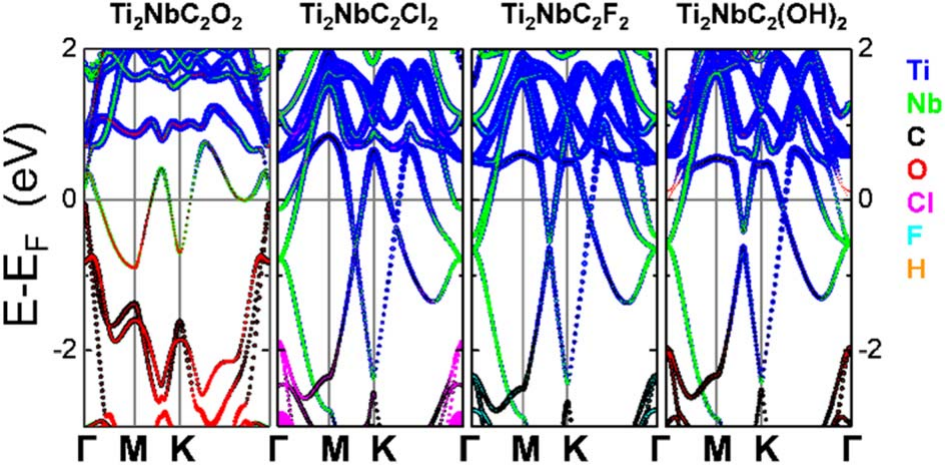
Band structure along the Γ–M–K–Γ path for the different MXenes functionalized.

Electronic properties are other essential characteristics of energy storage. Fig. 3 shows the band structure along the Γ–M–K–Γ pathway of functionalized systems. In all cases, the Fermi level is set as the energy reference. For all functionalized systems, a metallic behavior is noticed. Besides, the Cl- F- and OH-functionalized systems behave similarly around the Fermi level. The main contribution comes from the Ti orbitals, followed by the Nb and C orbitals. However, in the oxygen functionalized MXene, the main contribution around the Fermi level comes from the Nb orbitals, followed by the contribution of the O and Ti atoms. This change around the Fermi level is related to the strong interaction between Ti and O, which passivates the Ti atoms and modifies their electronic structure. To corroborate the electronic behavior of the MXenes, we also calculated the band structure employing the HSE06 hybrid functional, as we show in Fig. S3. Results confirm the metallic nature of the systems.

### 3.2 Ion intercalation process

Previous reports have demonstrated that ions only interact with the surface of the MXene,^27,28^*i.e.*, interstitial and substitutional metal ions result in instability. In contrast, adsorption over the functional groups is the only favorable interaction. Therefore, this work only considers the ion intercalation process as a surface phenomenon. The ion intercalation process is carried out by a systematic insertion of ions into the structure, where, in each step, the cell parameter is optimized; also, the vacuum space has been increased up to 20 Å to avoid interaction between periodic layers. See Fig. S4 for details. Since the structure has inversion symmetry, ion intercalation is carried out on both sides of MXenes. Different ions, such as Li, Na, K, Ca, and Mg, have been considered. Similar to the functionalization process, the ion insertion is investigated by considering three different high symmetry sites: Top′, T4′, and H3′, as shown in Fig. 2. Additionally, the formation energy formalism examines the stability of the structure. In our case, the formation energy is calculated by the following equation:^32^2
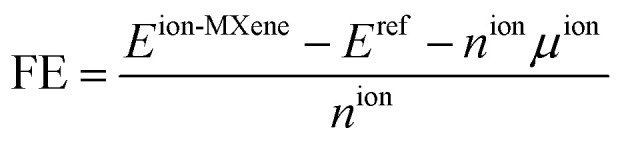
where *E*^ion-MXene^ is the energy of the MXene with *n* ions on the surface, *E*^ref^ is the energy of an arbitrary reference, in this case, the reference is the functionalized MXene without ions on its surface, *n*^ion^ is the number of ions in the system, and *μ*^ion^ is their chemical potential. With this definition, negative values indicate thermodynamic stability, while positive values indicate unstable systems. Table 2 summarizes the results for the first ion intercalation. About the Cl-functionalized MXene, T4′ site is the most stable configuration for Li and K intercalation, while the H3′ site is the stable one for Na intercalation; in the case of Ca and Mg ions, the intercalation process is unstable. In the case of the Ti_2_NbC_2_F_2_, the H3′ site is the most stable configuration for Li, Na, and K adsorption. However, Ca and Mg adsorption are unstable. The results for oxidized Ti_2_NbC_2_ MXene show that adsorption of the different ions considered in this work is favorable, with the H3′ site being the most stable configuration. Finally, in the Ti_2_NbC_2_(OH)_2_ MXene, only K adsorption is stable with the Top′ site as the most stable configuration.

**Table 2:** Formation energies (in eV) for the first ion intercalation

Ion	Ti_2_NbC_2_Cl_2_			Ti_2_NbC_2_F_2_			Ti_2_NbC_2_O_2_			Ti_2_NbC_2_(OH)_2_		
	Top′	T4′	H3′	Top′	T4′	H3′	Top′	T4′	H3′	Top′	T4′	H3′
Li	0.73	−0.26	−0.21	0.34	−0.38	−0.54	−1.06	−1.73	−2.01	0.68	0.41	0.12
Na	0.42	0.02	−0.01	0.15	−0.20	−0.26	−0.94	−1.56	−1.66	0.15	0.38	0.14
K	−0.20	−0.48	−0.46	−0.43	−0.70	−0.72	−1.49	−1.93	−1.98	−0.32	−0.05	−0.07
Ca	1.08	0.69	0.79	0.91	0.30	0.27	−0.77	−2.09	−2.43	0.97	0.69	0.54
Mg	1.39	1.33	1.34	1.30	1.55	1.29	0.47	−0.52	−1.00	1.09	0.96	0.96

### 3.3 Ion diffusion

**Fig. 4:**
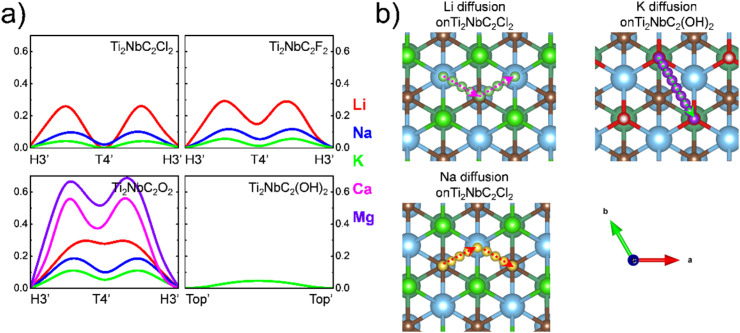
(a) Minimum energy pathway for the diffusion of the different metal ions considered in this work. (b) Atomistic representation of the three different pathways observed in the metal ion diffusion.

The ion diffusion across the surface is another essential characteristic for energy storage. Considering this, we investigated the minimum energy pathway (MEP) for the most stable adsorptions (see Table 2) from the most stable site to another equivalent position by employing CI-NEB calculations. Results are summarized in Fig. 4a. In all cases, the activation energy is the midpoint between two functional groups, serving as a bridge site. About Li diffusion on Cl-functionalized surface, the activation energy is 0.26 eV with H3′ as a metastable site; in the case of O-surface the activation energy is of the order of 0.30 eV, herein the T4′ site is the metastable configuration and H3′ is the stable site, similar to the diffusion of F surface with activation energy of 0.26 eV. In the case of Na ions, the diffusion along the Cl, O, and F surfaces is H3′–T4′–H3′ with activation energies of 0.10, 0.18, and 0.12 eV, respectively. In the case of K diffusion onto Ti_2_NbC_2_O_2_ and Ti_2_NbC_2_F_2_, H3′ is the most stable configuration while T4′ is the metastable site, the activation energies are 0.11 and 0.06 eV, respectively; opposite case is observed in Ti_2_NbC_2_Cl_2_ being T4′ site the most stable configuration and H3′ working as a metastable site, the activation energy is 0.04 eV; for the K diffusion onto OH-surface, Top′ site is the most stable configuration with activation energy of 0.05 eV. About Ca and Mg atoms, adsorption is only favorable onto the Ti_2_NbC_2_O_2_ MXene; in both cases, the H3′ and T4′ sites are the most stable and metastable sites, respectively; the activation energies for Ca and Mg are 0.55 and 0.66 eV, respectively. Fig. 3b shows the atomic models for the three different MEP observed from T4′ to T4′ (Li diffusion on Ti_2_NbC_2_Cl_2_), H3′ to H3′ (Na diffusion on Ti_2_NbC_2_Cl_2_), and from the Top′ to Top′ site (K diffusion on Ti_2_NbC_2_(OH)_2_). Notice a clear trend in the activation energies: in the alkali metal ions, the activation energy decreases as ion size increases; a similar trend is observed in alkaline earth metal ions. This behavior could be related to the ion size since large ion sizes reduce the interaction with the surface by reducing the energy difference between the different high symmetry sites. On the other hand, small ions increase these differences, as we show in Table S1 from the SI. Therefore, we can conclude that small ions are confined, limiting their mobility. Also, the activation energies for alkaline earth ions are larger than those for alkali ions; this is related to the oxidation state (OS) 2+ of Ca and Mg compared to the OS +1 of alkali ions.

### 3.4 Electrochemical performance

The electrochemical properties of the functionalized MXenes are investigated by calculating the Open Circuit Voltage (OCV) profile, considering the following reaction:

3 Ti_2_NbC_2_T_*x*_ + *n* × ion → ion_*n*_Ti_2_NbC_2_T_*x*_

where *n* is the number of ions present in the material, the following expression calculates the OCV:^32^4
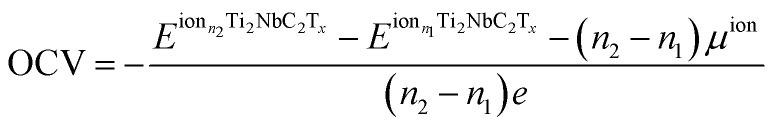


Being the 
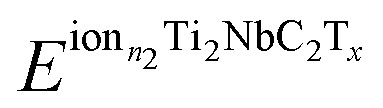
 and 
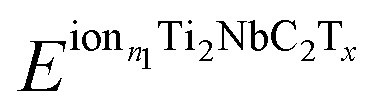
 the total energy of two different MXenes with different numbers of metal ions. *n*_2_ and *n*_1_ are the number of ions in the system, with *n*_2_ > *n*_1_. *μ*^ion^ is the chemical potential of the metal ions considered in this work, and *e* is the electron's charge. With this definition, positive values suggest a favorable intercalation process, while negative values suggest the formation of metal ion clusters, which interfere with the intercalation process. Therefore, the maximum number of ions that the anode can store is when the OCV drops to zero. The theoretical gravimetric capacity (*Q*) is calculated following the equation:5
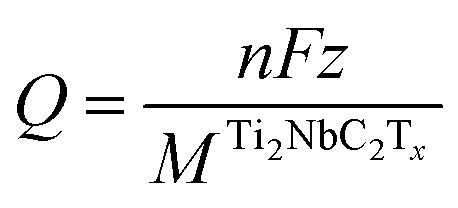
With *F* being the Faraday constant, *z* is the ion valence (+1 for alkali ions and +2 for alkaline earth ions), and *M*^Ti_2_NbC_2_T_*z*_^ is the atomic weight of the anode material.

**Fig. 5:**
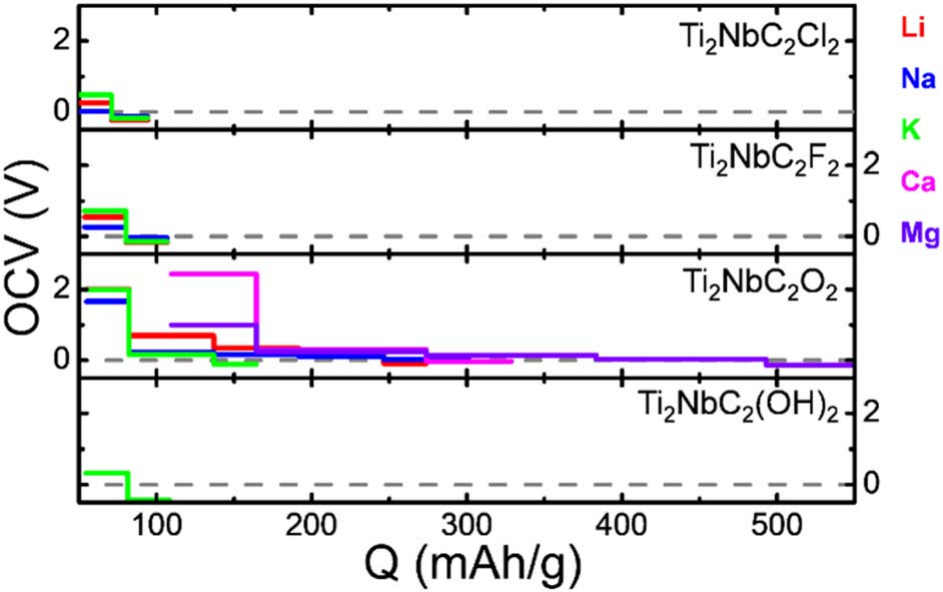
OCV curves as a function of theoretical gravimetric capacity.

Fig. 5 summarizes the results. Although Ti_2_NbC_2_Cl_2_ and Ti_2_NbC_2_F_2_ exhibit stable performance for Li, Na, and K ions, they have a low gravimetric capacity of less than 60 mAh g^−1^. Also, Li shows a maximum voltage of 0.54 V (0.26 V) in the Ti_2_NbC_2_F_2_ (Ti_2_NbC_2_Cl_2_) MXene. In the case of Na and K ions, the maximum voltages in the Cl-functionalized systems are 0.01 V and 0.50 V, respectively. At the same time, in the F-functionalized MXenes, they are 0.26 V, and 0.72 V. Similar behavior is noticed in the intercalation of K ions in the Ti_2_NbC_2_(OH)_2_ with a maximum capacity of 54 mAh g^−1^ and a maximum voltage of 0.32 V.

On the other hand, the oxidized MXene exhibits better results. A stable performance is observed with large Q values in all cases. Na exhibits the largest theoretical gravimetric capacity among the alkali ions, with 274 mAh g^−1^, followed by Li ions with 220 mAh g^−1^. In comparison, the K ions have a maximum capacity of 110 mAh g^−1^. Besides, Li and K have similar maximum voltage values of 2.01 and 1.98 V, respectively. Regarding alkaline earth ions, Mg provides the most significant *Q* values with a maximum capacity of 438 mAh g^−1^, while Ca reaches 219 mAh g^−1^. The maximum voltages obtained for Mg and Ca ions are 1 V and 2.43 V, respectively.

Our findings clearly show that oxidized MXene is the most promising candidate for use as an anode in metal ion batteries. Considering this, we focus on how the interaction between ions and the surface of MXene occurs.

### 3.5 Metal ion/Ti_2_NbC_2_O_2_ interaction

**Fig. 6:**
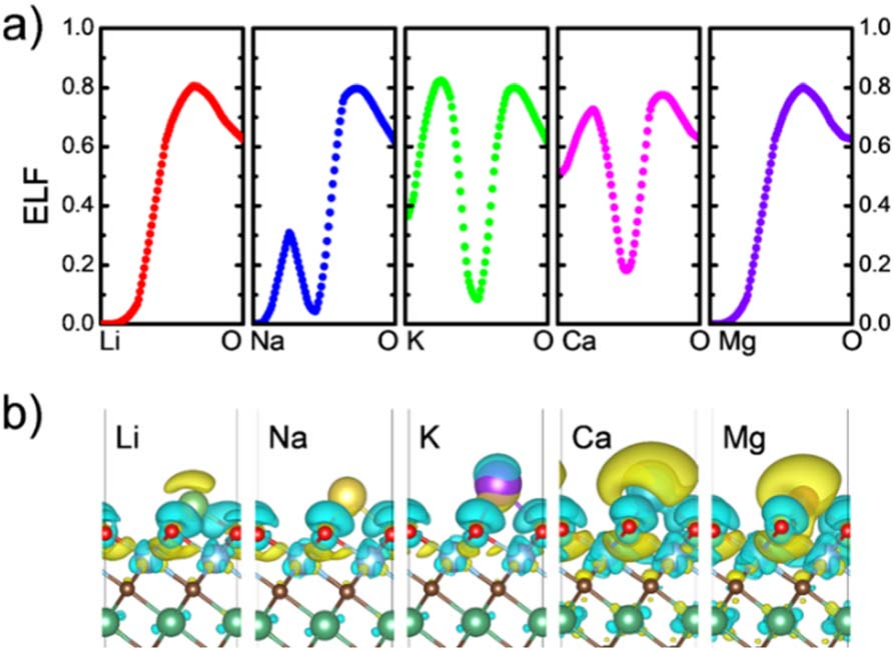
(a) ELF line profiles along the O-ion bond. (b) Charge density difference isosurfaces with an isovalue of 0.002 e Å^−3^: yellow and aqua colors represent charge accumulation and depletion, respectively.

To understand how the metal ions interact with the surface of the oxidized MXenes, we calculate the Bader charges. Our results show that Li and Na donate 0.87*e* to the substrate; in the case of K the ion donates 0.81*e*, slightly less than in previous cases. These results agree with Posysaev *et al.*^36^ and are associated with OS +1. About the alkaline earth ions, Ca donates 1.35*e*, while Mg donates 1.55*e* to MXenes; again, these values agree with Posysaev *et al.*^36^ and confirm an OS of +2. Fig. 6a depicts the Electron Localization Function (ELF) line profiles along the oxygen–metal ion bond. Notice that around the core region, the ELF decay, which is the typical behavior observed in pseudopotentials. Our finding reveals that in all cases, an ionic bond is formed. Notice that in the alkaline metal ions, the ionic nature of the bond decreases as the atomic number increases; this is related to the difference in electronegativities. Similar behavior is observed in alkaline earth metal ions.

We also investigate the charge transfer between metal ions and MXene by calculating the charge density difference defined as:

6 Δ*ρ* = *ρ*^complex^ − *ρ*^MXene^ − *ρ*^ion^

where *ρ*^complex^ is the charge density of the MXene with ions on the surface; *ρ*^MXene^ and *ρ*^ion^ are the charge densities of isolated MXene and ions, respectively. The Δ*ρ* for the different metal ions is shown in Fig. 6b with an isovalue of 0.002 e Å^−3^. The yellow isosurface is for charge accumulation, while the aqua isosurface is for charge depletion. We can observe that, in all cases, the metal ion transfers charge to the Ti_2_NbC_2_O_2_ MXene; the charge transfer is most notable in the Ca and Mg cases, which is expected since the oxidation state of these elements is +2. Besides, a charge depletion is noticed along the O-ion bond, which agrees with the ionic nature of the interaction.

**Fig. 7:**
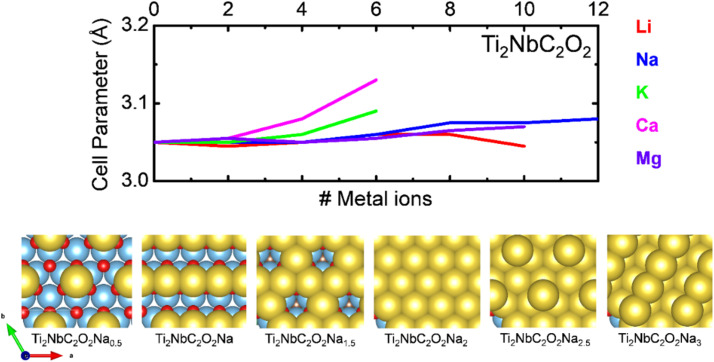
Evolution of the cell parameter as a function of the number of metal ions for the Ti_2_NbC_2_O_2_ MXene. The atomic arrangements for the Na intercalation process are shown.

As previously mentioned, the intercalation process is investigated by a systematic step-by-step metal ion insertion on both sides of the MXene. Where, in each step, the cell parameter is optimized. The upper panel of Fig. 7 shows the evolution of the cell parameter as the number of metal ions increases. Ti_2_NbC_2_O_2_ MXene has a cell parameter of 3.05 Å. In all cases, an expansion in the cell parameter is noticed (less than 3%), up to forming a complete monolayer on the surface (8 metal ions). Ca promotes the most intense expansion (2.45%), followed by K (1.31%). This behavior may be related to the ion size, affecting the battery's capacity. On the other hand, the expansion observed in Li, Na, and Mg cases is less than 1%. Once the first metal ion layer is formed, a second layer begins to form. In the case of Na and Mg, the expansion of the cell parameter continues with a maximum of 1% and 0.70%, respectively. Interestingly, in the case of Li, a contraction is observed going from an expansion of 0.33% when the full layer is formed to a contraction of −0.16%. This effect could be a consequence of the ion size.

The lower panel of Fig. 7 displays the atomic arrangement for the ion intercalation process. The figure only shows the Na case, since it is the system with the largest number of ions. However, all systems exhibit similar behavior. The first ion exhibits a three-fold coordination with oxygen atoms (Ti_2_NbC_2_O_2_Na_0.5_). Once the second intercalation is carried out (Ti_2_NbC_2_O_2_Na), the metal ions form chains along the [100] direction or [110] direction. In the third intercalation step (Ti_2_NbC_2_O_2_Na_1.5_), a hexagonal pattern is formed with a hollow in the center of the hexagon. In the four-step (Ti_2_NbC_2_O_2_Na_2_), the monolayer is completely formed. Once a complete monolayer is formed, a similar process occurs for the second layer, as seen in the Ti_2_NbC_2_O_2_Na_2.5_ and Ti_2_NbC_2_O_2_Na_3_ models.

Our findings demonstrate an excellent performance of the oxidized phase as an anode in metal-ion batteries. Table 3 compares our results with those of MXenes previously reported, considering the activation energies for ion diffusion along the surface of the MXene, the maximum *Q*, and the average OCV value. This last value is of importance, since values in the range between 0 and 1 V prevent dendrite formation, favoring the intercalation process.^37^ Unfortunately, K and Ca intercalations have average OCV values that fall outside the accurate range. Then, the intercalation process could be affected by dendrite formation, which in turn impacts the performance of the battery.

**Table 3:** Activation energies (in eV) for ion diffusion, maximum theoretical capacity (in mAh g^−1^), and average OCV values (in V) for different MXenes

Ion	MXene	Activation energy (eV)	*Q* (mAh g^−1^)	Average OCV (V)	Reference
Li	Ti_2_NS_2_	0.19	308.28	0.64	38
	V_2_NS_2_	0.17	299.52	0.82	38
	Ti_3_CNO_2_	0.26	269.00	1.00	39
	Nb_2_CO_2_	0.25	233.26	—	27
	Ti_2_NbC_2_O_2_	0.30	273.89	0.81	This work
Na	Ti_2_NS_2_	0.09	84.77	0.83	38
	V_2_NS_2_	0.09	99.80	0.53	38
	Ti_2_ZrC_2_O_2_	0.25	441.00	0.96	40
	TiZrCO_2_	0.27	586.00	0.83	40
	Ti_2_NbC_2_O_2_	0.18	219.11	0.43	This work
K	V_2_CO_2_	0.10	489.93	—	41
	V_2_CS_2_	0.06	200.24	—	41
	M_2_CO_2_ (M = Ti, Cr)	<0.10	>200	>1	42
	Ca_2_C	0.03	528.00	0.28	43
	Ti_2_NbC_2_O_2_	0.11	109.55	1.07	This work
Ca	V_2_N	1.14	770.80	1.18	44
	Sc_2_N	0.04	458.50	0.29	45
	Hf_3_C_2_F_2_	0.14	183.10	0.39	46
	Ti_2_NbC_2_O_2_	0.56	219.11	1.36	This work
Mg	Ti_2_CSO	0.71	524.54	0.19	47
	Ti_2_CSSe	0.47	230.45	0.21	47
	Mo_2_CO_2_	0.61	411.00	0.58	48
	Hf_3_C_2_F_2_	0.08	183.10	0.51	46
	Ti_2_NbC_2_O_2_	0.68	438.22	0.35	This work

About Li, notice that our MXene delivers a similar capacity to other MXenes with lower atomic weights, which suggests a better capacity for storing Li-ions. This fact makes Ti_2_NbC_2_O_2_ MXene a promising candidate for Li-ion batteries, offering lower activation energies and competitive OCV values. On the other hand, Na provides lower OCV values in comparison with previous reports, and the activation energies are lower than those of Ti_2_ZrC_2_O_2_ and TiZrCO_2_ MXenes. Although S-functionalized MXenes provide lower activation energies (90 meV), their capacities are under 100 mAh g^−1^, and their OCV values are larger than those of our oxidized MXenes. Therefore, the Ti_2_NbC_2_O_2_ MXene is a promising candidate for Na-ion batteries (NIBs). Similarly, our MXene offers larger capacities than Ti_2_CSSe and Hf_3_C_2_F_2_ MXenes for Mg-ions, also delivering similar values to low atomic weight MXenes (∼500 mAh g^−1^), denoting their remarkable capacity to store Mg-ions. The average OCV value is lower than that of Mo_2_CO_2_ and Hf_3_C_2_F_2_ MXenes, indicating their potential for implementation in Mg-ion batteries (MIBs).

About LIBs, carbonaceous electrodes such as graphite provide capacities of 372 mAh g^−1^, while B-doped graphene and graphene oxide deliver capacities of 415 and 419 mA g^−1^, respectively.^49^ On the other hand, different transition metal dichalcogenides (TMD) exhibit lower *Q* values below 100 mAh g^−1^.^50^ Monoelemental 2D layers such as phosphorene and boron sheets have been reported with theoretical capacities of 432 and 383 mAh g^−1^, respectively.^51,52^ In comparison, our MXene is under the graphite value, which could be related to the high molecular weight; however, the accurate average OCV value, stable performance, and lower diffusion barriers are of consideration for their implementation.

In the case of the NIBs, graphene provides a *Q* value of 308 mAh g^−1^.^49^ The doping of graphene with N, B, S, P, or F enhances the gravimetric capacity up to 384 mAh g^−1^.^49^ While the monolayer Ti_2_B_2_ has a *Q* = 342 mAh g^−1^.^51^ Similar to LIBs, our findings show values below graphite. Besides, considering a lower value of average OCV, the MXene may not be accurate for NIBs.

## 4. Conclusions

Our first-principles calculations analyzed the effect of different functional groups on the electrochemical performance of Ti_2_NbC_2_ MXene as an anode for alkali (Li, Na, and K) and alkaline earth (Ca and Mg) batteries. We analyzed the most common surface functional groups that appear on MXenes after chemical etching: O, Cl, F, and OH. The most stable site for the attached functional groups is H3, all functionalized systems present metallic characteristics, which are adequate for ion storage. The storage process in MXenes proceeds *via* ion intercalation, so we analyzed the electrochemical properties of the monolayers by calculating the open-circuit voltage as a function of the theoretical gravimetric capacities. The Cl- and F-functionalized MXenes have lower theoretical gravimetric capacities of ∼100 mAh g^−1^ for Li, Na, and K, while it is unstable for Ca and Mg. The OH-functionalized monolayer depicts instability and is not viable for energy storage applications. Finally, the O-functionalized MXene (Ti_2_NbC_2_O_2_) has excellent performance, with theoretical gravimetric capacities of 274, 219, and 438 mAh g^−1^ for Li, Na, and Mg, respectively, which evidences good capacity to store alkali and alkaline earth metal ions. Our results establish Ti_2_NbC_2_O_2_ as a competitive MXene material, with a comparable storage capacity to other materials reported in the literature for Li and Mg metal ions.

## Author contributions

R. Ponce Perez: conceptualization, formal analysis, investigation, methodology. J. Guerrero Sanchez: data curation, formal analysis, conceptualization, funding acquisition. M. G. Moreno Armenta: project administration, funding acquisition, data curation, formal analysis.

## Conflicts of interest

There are no conflicts to declare.

## Supplementary Material

RA-015-D5RA04549E-s001

## Data Availability

The data supporting this article have been included as part of the SI. Optimization curves; structural models of the MXenes in A/B/A/B/A stackings; band structure with HSE06 functional; planar average potential; relative energies (in eV) for the different high symmetry sites. See DOI: https://doi.org/10.1039/d5ra04549e.
