# Supplementary material for: Bimetallic Ti2NbC2 MXene as anode material for metal ion batteries: influence of functional groups
Source: RSC Adv. 2025 Aug 26;15(37):30156–64. doi: 10.1039/d5ra04549e (PMC12378756; doi:10.1039/d5ra04549e)
Supplement: RA-015-D5RA04549E-s001 [file RA-015-D5RA04549E-s001.pdf]

Supplementary Information (SI) for RSC Advances.
This journal is © The Royal Society of Chemistry 2025

Supplementary Information

Bimetallic Ti_2NbC_2 MXene as anode material for metal ion batteries: influence of functional groups

Rodrigo Ponce Perez,[a] Jonathan Guerrero Sanchez,[a] and Maria G. Moreno Armenta*[a]*

[a] Centro de Nanociencias y Nanotecnología, Universidad Nacional Autónoma de México, Ensenada, B.C, CP 22800, México.

** Corresponding authors email: R.P.P. rponce@ens.cnyn.unam.mx; M.G.M.A moreno@ens.cnyn.unam.mx*

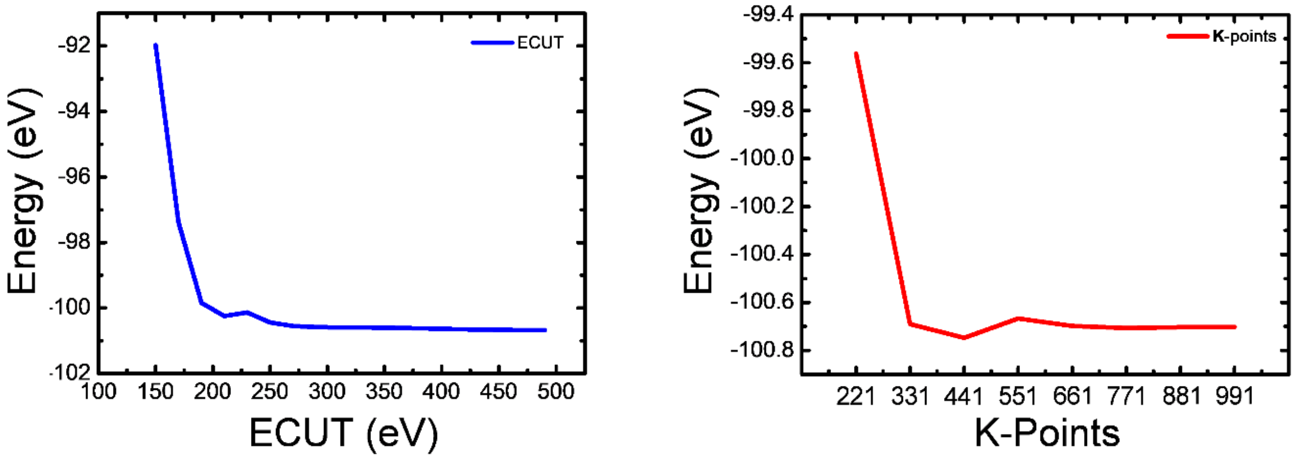

Figure S1. Optimization curves for computational parameters, the left panel is for the energy cutoff (ECUT); the right panel is for K-points optimization.

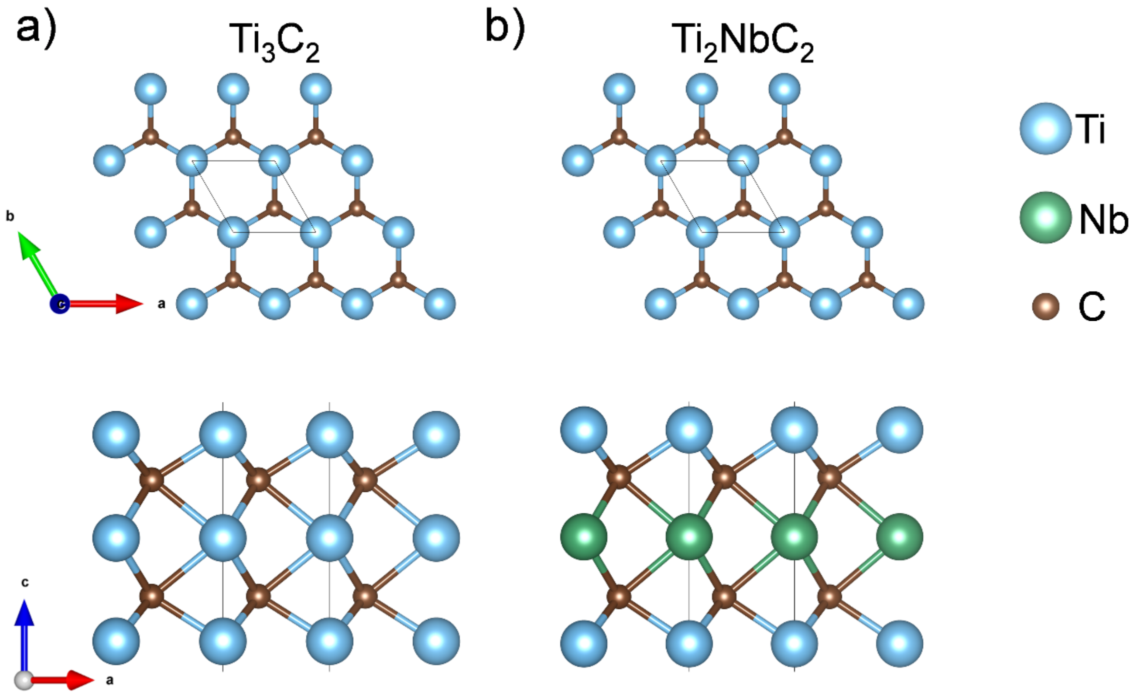

Figure S2. Top and Side views of a) Ti_3C_2 and b) Ti_2NbC_2 MXenes in the A/B/A/BA stacking.

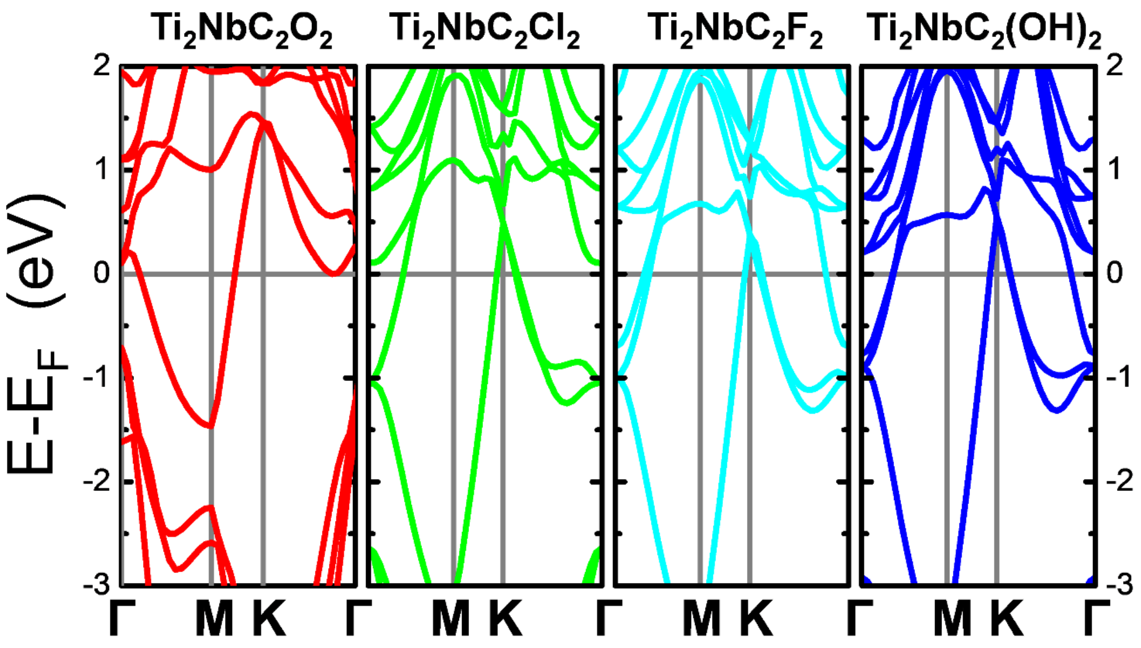

Figure S3. Band structure along the **Γ-M-K-Γ** path for the different MXenes functionalized employing the HSE06 functional.

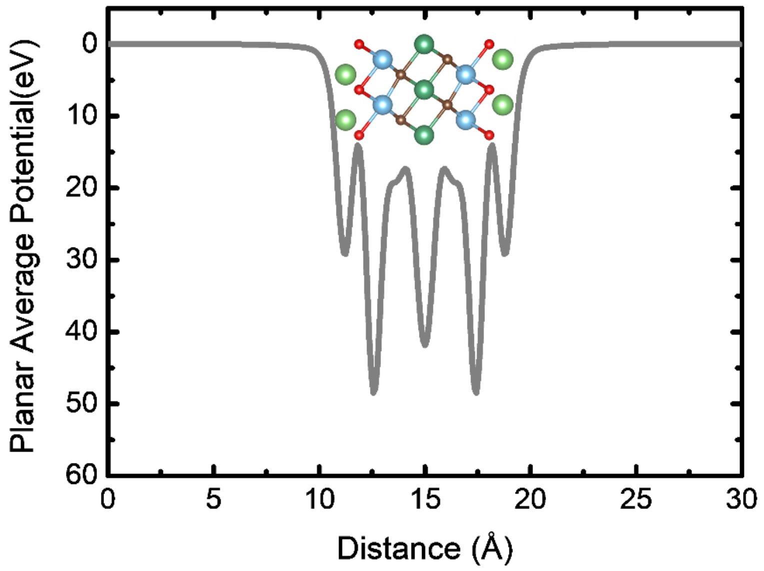

Figure S4. Planar average potential for the $Ti_2NbC_2O_2$ MXenes with a full monolayer of Li.

Table S1. Relative energies (in eV) between high symmetry sites according to the values from **Table 2**. Only stable values are included.

Ion	$Ti_2NbC_2Cl_2$			$Ti_2NbC_2F_2$			$Ti_2NbC_2O_2$			$Ti_2NbC_2(OH)_2$		
	Top'	T4'	H3'	Top'	T4'	H3'	Top'	T4'	H3'	Top'	T4'	H3'
Li	0.99	0.00	0.05	0.88	0.16	0.00	0.95	0.28	0.00	-	-	-
Na	0.43	0.03	0.00	0.41	0.06	0.00	0.72	0.10	0.00	-	-	-
K	0.28	0.00	0.02	0.29	0.02	0.00	0.49	0.05	0.00	0.00	0.27	0.25
Ca	-	-	-	-	-	-	1.66	0.34	0.00	-	-	-
Mg	-	-	-	-	-	-	1.47	0.48	0.00	-	-	-
